# Burden of Medical Costs Associated with Severe Maternal Morbidity in South Korea

**DOI:** 10.3390/healthcare12232414

**Published:** 2024-12-02

**Authors:** Jin Young Nam, Soojeong Shim

**Affiliations:** Department of Healthcare Management, Eulji University, Sungnam 13135, Gyeonggi-do, Republic of Korea

**Keywords:** severe maternal morbidity, medical costs, pregnancy-related medical costs, blood transfusion, NHIS cohort

## Abstract

Background: Adverse maternal health outcomes lead to health loss and unnecessary medical costs. However, few have explored how severe maternal morbidity (SMM) affects medical costs separately from blood transfusion. Therefore, the aim of this study was to evaluate the delivery-related costs of healthcare services in patients with and without SMM as well as blood transfusion. Methods: This retrospective cohort study used the National Health Insurance Service (NHIS) Delivery Cohort database in South Korea. We included all delivering mothers in South Korea from 2016 to 2021, except those with incomplete data, totaling 1,517,773 participants. The measured outcomes included delivery-related medical costs associated with SMM. A generalized estimating equation model with a log link, gamma distribution, and robust standard errors was used to estimate the mean delivery-related medical costs of SMM. Results: SMM occurred in 2.2% of the cohort. The adjusted mean delivery-related medical costs were approximately 2.1- and 1.4-fold higher in cases with SMM without blood transfusion and only blood transfusion than in those without SMM, respectively ($2005, 95% CI: $1934–2078 and $1339, 95% CI: 1325–1354, respectively). The adjusted mean delivery-related medical costs were 1.5-fold higher in cases with SMM with blood transfusion than in those without SMM (SMM $1539, 95% CI: $1513–$1565). Conclusions: Medical costs associated with delivery-related SMM with or without blood transfusion were significantly higher than those of normal deliveries, with excess costs varying according to existing healthcare policies. Policymakers should consider supporting programs to prevent high medical costs by improving maternal health.

## 1. Background

Severe maternal morbidity (SMM) is a major maternal complication defined by the Centers for Disease Control and Prevention (CDC) as an unexpected outcome of childbirth that results in short- or long-term health consequences [1]. The CDC defines SMM as encompassing indicators based on diagnoses and procedures, such as disseminated intravascular coagulation, shock, hysterectomy, and blood transfusion [2]. The incidence of SMM has increased over the last decade, with approximately 50,000–60,000 women per year experiencing severe, potentially life-threatening conditions during pregnancy and delivery in the United States [2]. More recently, the CDC has recommended that blood transfusions be excluded from SMM indicators, but states that they could be examined separately [3] because of their validity [4]. Efforts to improve maternal health outcomes have shown that SMM is a precursor to maternal mortality, and the California Maternal Quality Care Collaboration has shown that the reduction of maternal mortality rates is possible [5].

Globally, there are approximately 131 million newborns annually [6], with childbirth representing the most common cause of hospitalization in developed countries [5]. South Korea has the lowest fertility rate worldwide, but had total hospital costs of approximately 540 million dollars for maternal childbirth-related medical costs in 2022 [7]. As this cost included only insurance-covered payments, the real-world cost volume may be considerably more when uncovered health services are included. Although the occurrence of SMM during delivery admission is rare, such circumstances can result in increased hospital expenses because of the requirement for extra procedures and prolonged hospital stays [1]. This imposes a disproportionate economic strain on the national population of women giving birth.

Despite growing attention, limited information on the economic burden of maternal mortality and morbidity is available [8]. Highlighting aspects of this economic burden could reveal the extent of these public health problems [9]. However, studies on the effect of SMM on costs are limited [5,8,10,11,12]. Existing studies have shown that SMM increases costs [5,8,10,11,12]; however, most have focused on the U.S. population [5,8,10,11], with only one report from an Asian country [12]. Moreover, some previous studies included only a small SMM sample [11], others excluded physician costs [8,10], and others reported 10–20-year-old data [12]. Furthermore, only one study investigated the cost of SMM separately from that of blood transfusion [5]. Finally, no previous study has compared direct medical costs associated with overall SMM, SMM with or without blood transfusion, or only blood transfusion, nationwide over an extended period.

Therefore, we examined the association of SMM and SMM with or without blood transfusion with childbirth-related medical costs in South Korea using a nationwide population-based delivery cohort database. In this way, it seeks to provide insights into the economic burden of maternal morbidity and inform strategies to optimize maternal healthcare policies.

## 2. Methods

### 2.1. Data Source and Study Population

This population-based study used the database of the Korean National Health Insurance Service (NHIS), the single insurer for the entire country’s population, in which approximately 98% of all South Koreans are enrolled [13]. The NHIS database retains data on sociodemographic characteristics, healthcare use (received services and treatment costs), clinically determined diagnostic codes from the International Classification of Diseases (ICD), 10th revision, and prescriptions with drug codes, days prescribed, and daily dosages [13]. The database uses de-identified join keys to link databases while ensuring patient anonymity [13]. The study design was reviewed and approved by our Institutional Review Board (IRB Number: EU22-27). The requirement for informed consent was waived given that the data did not contain any identifiable information.

We extracted data from the NHIS claims database for all women who delivered at medical institutions in South Korea between 1 January 2008 and 31 December 2021; however, we excluded patients before 30 June 2016, in alignment with the changes in medical cost policies (see Supplementary File S1). Childbirth was identified using all inpatient records, including pregnancy-related diagnosis and vaginal or cesarean delivery procedure codes. The study population included women aged 15–49 years and those who gave birth until 19 November 2021, such that the data on childbirth within 6 weeks postpartum could be analyzed. The total population included 5,152,522 deliveries between 1 January 2008 and 31 December 2021. Notably, we excluded women who gave birth after 19 November 2021 (*n* = 7415), had no healthcare institution delivery data (*n* = 7836), or those who gave birth before 30 June 2016 (*n* = 3,619,498). In total, 1,517,773 deliveries from 1 July 2016 to 19 November 2021 were included in this study.

### 2.2. Delivery-Related Medical Costs

Delivery-related medical costs were calculated from the claimed total direct costs during the delivery hospitalization and 6-week postpartum period. As the NHIS database excludes outpatient drug costs or uncovered healthcare service costs (such as uncovered treatments, medical administrations and injections, and nonstandard accommodations), the costs reported herein do not include those of uncovered services.

To compare prices from different calendar years, costs were inflated to 2020 values using the South Korea Consumer Price Index for healthcare from the Bank of Korea by multiplying them by a year-specific inflation factor [14]. To express costs in U.S. dollars, they were converted from Korean won to U.S. dollars using the exchange rates of the Ministry of Economy and Finance for each year [15].

### 2.3. Severe Maternal Morbidity

Women with SMM were defined as having at least 1 of the 21 potentially life-threatening maternal conditions or adverse events identified by the CDC as indicators of SMM [1,16] using ICD codes, including the new ICD version used from October 2015 onward, which contained blood transfusion [3] (see Supplementary File S3). In addition, we adopted a more constrained definition of SMM, segregating instances where the sole criterion for SMM was a maternal blood transfusion (referred to as “blood transfusion only cases”), thus delineating subcategories for cases involving blood transfusion alone and those encompassing SMM with indications other than blood transfusion.

### 2.4. Covariates

The covariates were maternal sociodemographic and clinical factors. The sociodemographic factors included maternal age (<19 years, 19–24 years, 25–29 years, 30–34 years, 35–39 years, 40–44 years, or >45 years); income level (quartile); type of insurance (self-employed insured, employee insured, or medical aid); and residential area (Seoul, metropolitan areas, small cities, or rural areas). Maternal clinical factors included mode of delivery (spontaneous vaginal delivery, instrumental delivery, or cesarean section delivery); preterm birth (delivered at <37 vs. ≥37 weeks); parity (nulliparous or multiparous); multiple birth status (singleton vs. twin or more); adequacy of prenatal care (estimated by the Kessner Adequacy of Prenatal Care Index [17], which categorized adequate vs. inadequate, including intermediate, prenatal care); obstetric comorbidity (assessed by Bateman’s obstetric comorbidity index [18]); and delivery year. Supplementary File S2 provides a detailed explanation of all variables.

### 2.5. Statistical Analyses

We analyzed the distributions of maternal mortality and SMM according to maternal sociodemographic and clinical factors using descriptive statistics. We calculated the unadjusted mean delivery-related medical costs and their 95% confidence intervals (CIs) to test their differences for childbirth with and without maternal mortality and SMM and for all variables using the Kruskal–Wallis test. We used a generalized estimating equation (GEE) model with a log link, a gamma distribution, and robust standard errors to estimate the mean delivery-related medical costs of SMM and other variables adjusted for covariates. We performed a stratified analysis using the GEE model to calculate the association between adjusted delivery-related medical costs and SMM by maternity residential area. All statistical analyses were conducted using SAS 9.4 (SAS Institute, Inc., Cary, NC, USA). The level of significance was set at *p* < 0.05.

## 3. Results

Of the 1,517,773 deliveries included in this study (Table 1), SMM occurred in 2.2% of the participants, SMM without blood transfusion occurred in approximately 0.7%, and only blood transfusion occurred in 1.5% (Table 1).

The average total delivery-related medical cost for all participants was $1723 (95% CI: $1722–$1725) (Table 2). In cases with SMM, the cost was $12,725 (95% CI: $8872–$16,558), while in those without SMM, the cost was $1697 (95% CI: $1696–$1698). In the subcategory model, the cost of SMM without blood transfusion was $3731 (95% CI: $3606–$3857), and the cost of blood transfusion only was $2515 (95% CI: $2493–$2536). The average medical costs varied significantly based on the covariates (*p* < 0.0001 for each) (Table 2).

The mean delivery-related total medical costs were adjusted for all covariates, and the association of the adjusted costs with SMM was analyzed (Table 3). Regarding SMM without blood transfusion and blood transfusion only, the adjusted mean (CI) delivery-related costs were $2005 ($1934–$2078) and $1339 ($1325–$1354), respectively, representing 2.1- and 1.4-fold higher costs than those without SMM, respectively (Table 3).

The total delivery-related medical cost for all women with SMM without blood transfusion or blood transfusion only (number with SMM × adjusted mean cost of SMM) was approximately $20.6 million and $32.0 million, respectively, representing 13.5% and 21.0% of the costs in those models (Table 3).

## 4. Discussion

This large, population-based delivery cohort study estimated the association of SMM with delivery-related medical costs in South Korea. SMM without blood transfusion had more than double the costs of delivery; those of blood transfusion were 1.4 times higher than those without SMM after adjusting for covariates. As a result, more than $20 million was spent from 2016 to 2021, which is more than the normal delivery costs for SMM. Importantly, approximately 52% of SMM without blood transfusion-, 28% of blood transfusion only-, and 37% of overall SMM delivery-related medical costs could be avoided by preventing these conditions. Our findings indicate that SMM cases impose a considerable economic burden on the healthcare system, with an estimated $20.6 million associated with preventable conditions from 2016 to 2021. This highlights the need for cost-effective interventions aimed at improving prenatal care and reducing SMM-related complications.

Previous studies have shown similar results for the association between SMM and medical costs. Phibbs et al. reported that SMM correlated with a 60% increase in hospital costs [5]. Chen et al. showed that women with SMM in 2011–2012 had a 2.1-fold higher mean cost of delivery hospitalization than those without SMM in the U.S. National Inpatient Sample [10]. Nam et al. showed that women with SMM in South Korea (2003–2013) incurred a 1.7-fold higher mean expense for delivery care than those without SMM, based on the National Health Insurance Service National (NHIS) Sample Cohort [12], which is similar to our result. Notably, our study showed that SMM increased the total medical costs by approximately 37%, a result on the lower end of the range of reported results. Several studies have shown that SMM costs differ depending on the inclusion of blood transfusion. Debbink et al. reported that SMM events according to CDC criteria with blood transfusion were associated with a 2.45-fold cost increase compared to uncomplicated deliveries, whereas excluding transfusions led to a 3.26-fold increase [19]. Phibbs et al. SMM cases had 23% higher costs, which increased to 29% when cases with blood transfusion as the only indication were excluded [20].

The reason for the lower delivery-related medical costs compared to that in the previous study may be explained by the inclusion of blood transfusion as an event defining SMM regardless of the number of units administered, as in the CDC’s algorithm. Most patients were considered to have SMM solely based on receiving a transfusion with only 1–2 units of blood products, despite lacking any other serious adverse events, and incurred only half of the hospital costs for childbirth compared to patients with SMM for non-transfusion reasons [5]. Given that blood transfusion accounted for >77% of SMM cases in South Korean women in a previous study [21] and given that our study included blood transfusion as a defining factor for SMM, we likely included patients who received only transfusions and did not have serious adverse events, thereby attenuating the medical costs in the SMM group. Moreover, as this database only included insurance-covered procedures, the costs might be underestimated. Therefore, the observed difference in medical costs depending on the inclusion or exclusion of blood transfusion in SMM cases can be attributed to the fact that the inclusion of transfusion-only cases dilutes the overall cost severity. This occurs because these cases often involve simple transfusions rather than procedures associated with higher severity of morbidity.

In South Korea, recent demographic and healthcare trends highlight significant changes in childbirth patterns and their implications for maternal health. First, childbirth trends indicate a growing proportion of high-risk pregnancies. According to the 2023 Korean Statistical Information Service (KOSIS) data, the average maternal age for first births was approximately 33 years [22]. Births among women aged 35 years or older have increased, along with a rise in multiple pregnancies due to the inclusion of assisted reproductive technology (ART) in national health insurance [23]. These trends align with international criteria for high-risk pregnancies and contribute to an increased risk of SMM. Second, these changes have driven a preference for cesarean section (C-section) deliveries. Data from the KOSIS show that the cesarean section rate in South Korea has steadily risen, surpassing 60% in 2022 [24]. According to OECD data, South Korea ranks second among OECD countries in C-section rates [25]. Therefore, the increasing preference for cesarean sections has led to higher risks of complications [26], such as infections and hemorrhage, along with significantly increased healthcare costs.

This study has several strengths. First, to the best of our knowledge, no other investigation has estimated the association of SMM with or without blood transfusion with delivery-related direct medical costs using a nationally representative database that included all delivering women in South Korea during an extended recent year. While several studies have examined the relationship between delivery costs and one of these conditions, none have estimated the costs in relation to SMM separately from that of blood transfusion. Second, the endpoints were adjusted for numerous demographic and obstetric co-varying factors, allowing the detection of significant differences in the diverse case mix. Third, this study provides considerable data to support future studies on not only the association between maternal health outcomes and medical costs but also the disease burden of maternal mortality and morbidity in various segments of the delivery population.

This study also has some limitations. First, because the NHIS delivery cohort database excludes information on healthcare services not covered by insurance; given that policies affecting covered services changed during the 5-year span of the study, some costs were inconsistently captured. For instance, coverage of the specialist medical service fee changed in January 2018, and coverage of some nonstandard hospital accommodations changed in July 2019. As a result, the total medical costs may have been underestimated. Second, the database excluded uncovered procedures and prescriptions, such as the hospital private room fee or the fee for using a new drug; therefore, the cost might be underestimated. Third, because the NHIS Delivery Cohort database uses revised ICD-10 codes that exclude procedure codes, we converted ICD-10 codes for procedures such as cardiac rhythm conversion, blood transfusion, hysterectomy, and ventilation to electronic data interchange claims codes, which may have made the identification of procedure-defined SMM cases less accurate.

## 5. Conclusions

Women with SMM were associated with excessive delivery-related medical costs compared to those without SMM. Approximately 52% of those related to SMM without blood transfusion and 27% of those related to only blood transfusion (representing more than $20 million over 5 years) are potentially preventable. These findings highlight the economic burden of SMM and the importance of targeted interventions, such as improving prenatal care, promoting alternative treatments like high-dose intravenous iron to reduce the need for blood transfusions, and addressing demographic trends contributing to high-risk pregnancies. Policymakers should prioritize cost-effective strategies, including expanding insurance coverage for preventive maternal health services, to reduce both the incidence and financial impact of SMM and support sustainable maternal health outcomes.

## Figures and Tables

**Table 1:** General characteristics of study population.

	Severe Maternal Morbidity					
	No		Yes		Total	
	n	(%)	n	(%)	n	(%)
Total	1,517,773	(97.8)	34,146	(2.2)	1,551,919	(100.0)
Maternal age						
<19	3292	(0.2)	131	(0.4)	3423	(0.2)
19–24	52,218	(3.4)	1152	(3.4)	53,370	(3.4)
25–29	267,304	(17.6)	4922	(14.4)	272,226	(17.5)
30–34	668,406	(44.0)	13,322	(39.0)	681,728	(43.9)
35–39	447,572	(29.5)	11,703	(34.3)	459,275	(29.6)
40–44	76,479	(5.0)	2769	(8.1)	79,248	(5.1)
45+	2502	(0.2)	147	(0.4)	2649	(0.2)
Income level						
1Q	316,484	(20.9)	7409	(21.7)	323,893	(20.9)
2Q	347,982	(22.9)	7589	(22.2)	355,571	(22.9)
3Q	530,032	(34.9)	11,717	(34.3)	541,749	(34.9)
4Q	323,275	(21.3)	7431	(21.8)	330,706	(21.3)
Type of insurance						
Self-employed insured	300,903	(19.8)	7210	(21.1)	308,113	(19.9)
Employee insured	1,207,751	(79.6)	26,427	(77.4)	1,234,178	(79.5)
Medical aid	9119	(0.6)	509	(1.5)	9628	(0.6)
Residential area						
Seoul	288,494	(19.0)	6471	(19.0)	294,965	(19.0)
Metropolitans	391,657	(25.8)	8510	(24.9)	400,167	(25.8)
Small cities	759,611	(50.1)	17,175	(50.3)	776,786	(50.1)
Rural areas	78,011	(5.1)	1990	(5.8)	80,001	(5.2)
Mode of delivery						
Spontaneous vaginal delivery	341,814	(22.5)	4439	(13.0)	346,253	(22.3)
Instrumental delivery	464,053	(30.6)	9981	(29.2)	474,034	(30.6)
Cesarean section delivery	711,906	(46.9)	19,726	(57.8)	731,632	(47.1)
Preterm birth						
No	1,466,320	(96.6)	30,185	(88.4)	1,496,505	(96.4)
Yes	51,453	(3.4)	3961	(11.6)	55,414	(3.6)
Prenatal care						
Adequate	1,408,918	(92.8)	31,289	(91.6)	1,440,207	(92.8)
Inadequate	108,855	(7.2)	2857	(8.4)	111,712	(7.2)
Parity						
Nulliparous	815,808	(53.8)	20,285	(59.4)	836,093	(53.9)
Multiparous	701,965	(46.3)	13,861	(40.6)	715,826	(46.1)
Multiple birth status						
Singleton	1,489,886	(98.2)	31,544	(92.4)	1,521,430	(98.0)
Twin or more	27,887	(1.8)	2602	(7.6)	30,489	(2.0)
Obstetric comorbidity						
0	882,680	(58.2)	14,503	(42.5)	897,183	(57.8)
1+	635,093	(41.8)	19,643	(57.5)	654,736	(42.2)
Year						
2016	171,520	(11.3)	3755	(11.0)	175,275	(11.3)
2017	319,638	(21.1)	6812	(20.0)	326,450	(21.0)
2018	292,052	(19.2)	6531	(19.1)	298,583	(19.2)
2019	270,342	(17.8)	5896	(17.3)	276,238	(17.8)
2020	240,860	(15.9)	5280	(15.5)	246,140	(15.9)
2021	223,361	(14.7)	5872	(17.2)	229,233	(14.8)

**Table 2:** Unadjusted mean costs for total delivery-related medical costs in relation to socioeconomic and obstetric factors.

	Delivery-Related Medical Costs				
	N	Mean Cost (USD)	Lower 95% CI (USD)	Upper 95% CI (USD)	*p*-Value
Total	1,551,919	1723	1722	1725	<0.0001
Severe maternal morbidity					
No	1,517,773	1697	1696	1698	
SMM without blood transfusion	10,253	3731	3606	3857	
Only blood transfusion	23,893	2515	2493	2536	
Maternal age					<0.0001
<19	3423	1559	1531	1587	
19–24	53,370	1601	1595	1606	
25–29	272,226	1639	1636	1642	
30–34	681,728	1674	1672	1676	
35–39	459,275	1815	1812	1818	
40–44	79,248	1990	1982	1998	
45+	2649	2093	2051	2135	
Income level					<0.0001
1Q	323,893	1716	1713	1719	
2Q	355,571	1726	1723	1729	
3Q	541,749	1720	1718	1723	
4Q	330,706	1733	1730	1736	
Type of insurance					<0.0001
Self-employed insured	308,113	1736	1733	1739	
Employee insured	1,234,178	1723	1721	1724	
Medical aid	9628	1458	1442	1475	
Residential area					<0.0001
Seoul	294,965	1767	1764	1770	
Metropolitans	400,167	1721	1718	1723	
Small cities	776,786	1707	1705	1709	
Rural areas	80,001	1737	1732	1743	
Mode of delivery					<0.0001
Spontaneous vaginal delivery	346,253	1325	1323	1327	
Instrumental delivery	474,034	1489	1487	1491	
Cesarean section delivery	731,632	2064	2062	2067	
Preterm birth					<0.0001
No	1,496,505	1709	1708	1710	
Yes	55,414	2110	2098	2123	
Prenatal care					<0.0001
Adequate	1,440,207	1705	1703	1706	
Inadequate	111,712	1968	1962	1974	
Parity					<0.0001
Nulliparous	836,093	1803	1802	1805	
Multiparous	715,826	1630	1628	1632	
Multiple birth status					<0.0001
Singleton	1,521,430	1709	1708	1710	
Twin or more	30,489	2440	2426	2454	
Obstetric comorbidities					<0.0001
0	897,183	1628	1627	1630	
1+	654,736	1854	1851	1856	
Year					<0.0001
2016	175,275	1278	1275	1282	
2017	326,450	1537	1534	1539	
2018	298,583	1637	1634	1640	
2019	276,238	1754	1751	1758	
2020	246,140	2064	2060	2067	
2021	229,233	2040	2037	2044	

CI, confidence interval; USD, US dollars. Tested for significant differences in mean costs by characteristics using the Kruskal–Wallis test for nonparametric outcomes.

**Table 3:** Association between delivery-related medical costs and severe maternal morbidity adjusted for all covariates.

	Delivery-Related Medical Costs			
	Mean Cost (USD)	Lower 95% CI (USD)	Upper 95% CI (USD)	*p*-Value
Intercept	969	967	972	<0.0001
Severe maternal morbidity				
No				
SMM without blood transfusion	2005	1934	2078	<0.0001
Only blood transfusion	1339	1325	1354	<0.0001
Maternal age				
<19	979	965	994	0.0875
19–24	970	965	976	0.3823
25–29				
30–34	975	971	979	<0.0001
35–39	997	991	1003	<0.0001
40–44	1028	1021	1035	<0.0001
45+	1048	1030	1067	<0.0001
Income level				
1Q	967	963	971	0.0033
2Q	965	961	969	<0.0001
3Q	964	961	968	<0.0001
4Q				
Type of insurance				
Self-employed insured	967	963	971	0.0036
Employee insured				
Medical aid	842	833	852	<0.0001
Residential area				
Seoul				
Metropolitans	952	948	956	<0.0001
Small cities	942	939	946	<0.0001
Rural areas	969	964	974	0.9539
Mode of delivery				
Spontaneous vaginal delivery				
Instrumental delivery	1056	1052	1060	<0.0001
Cesarean section delivery	1427	1422	1433	<0.0001
Preterm birth				
No				
Yes	1092	1084	1100	<0.0001
Prenatal care				
Adequate				
Inadequate	959	955	964	<0.0001
Parity				
Nulliparous	1046	1043	1050	<0.0001
Multiparous				
Multiple birth status				
Singleton				
Twin or more	1061	1053	1069	<0.0001
Obstetric comorbidity				
0				
1+	1012	1006	1018	<0.0001
Year				
2016				
2017	1157	1151	1162	<0.0001
2018	1223	1217	1228	<0.0001
2019	1299	1293	1306	<0.0001
2020	1517	1510	1524	<0.0001
2021	1482	1475	1489	<0.0001

CI, confidence interval; USD, US dollars. Adjusted for maternal age, income level, type of insurance, residential area, mode of delivery, preterm birth, prenatal care, parity, multiple birth status, obstetric comorbidity, and delivery year.

## Data Availability

No data are available because the National Health Information Database can only be accessed by researchers authorized by the Korean National Health Insurance Service.

## Supplementary Materials

The following supporting information can be downloaded at: https://www.mdpi.com/article/10.3390/healthcare12232414/s1, File S1: Alignment of model periods with timing of Korean health policy changes; File S2: The explanation of variables; File S3: The codes of Severe Maternal Morbidity as defined and identified by the CDC.

## List of Abbreviations

CDC	Centers for Disease Control and Prevention
SMM	severe maternal morbidity
NHIS	National Health Insurance Service
ICD	International Classification of Diseases
CI	confidence interval
GEE	a generalized estimating equation
KOSIS	Korean Statistical Information Service
C-SECTION	cesarean section
