# Supplementary material for: Burden of Medical Costs Associated with Severe Maternal Morbidity in South Korea
Source: Healthcare (Basel). 2024 Dec 2;12(23):2414. doi: 10.3390/healthcare12232414 (PMC11641367; doi:10.3390/healthcare12232414)
Supplement: Supplementary file 1 [file healthcare-12-02414-s001.zip › healthcare-3289900-supplementary.pdf]

Supplementary file S1. Alignment of model periods with timing of Korean health policy changes

We chose the study periods according to the timing of Korean government policy changes regarding fee-for-service coverage for cesarean section delivery. Korea's health insurance fee system previously used a resource-based, relative-value fee-for-service approach. To address issues with the fee-for-service system, the Ministry of Health and Welfare introduced a diagnosis-related group (DRG)-based prospective payment system in September 2003, establishing seven disease groups (revision surgery, tonsil surgery, anal surgery, hernia surgery, appendectomy, uterine surgery, and cesarean section). In July 2012, this approach was universally applied to hospitals and clinics, and in July 2013, it was expanded to include medical institutions at and above the comprehensive hospital level.

To encourage the expansion of healthcare coverage, the Korean government has supported several fertility-related policies. Spontaneous vaginal delivery has been free of charge to the patient since January 2005. In the four years from its universal adoption for prospective payment in July 2012 to June 2016, cesarean section delivery accounted for 20% of the total out-of-pocket costs paid. Since July 2016, it has represented 5% of total delivery-related costs. In alignment with these changes, we excluded before June 30, 2016 because of the changes of medical cost policies. Therefore, we chose the data from July 1, 2016, to November 19, 2021 because of inclusion of the 42-day postpartum period for each delivery examined in the study.

Supplementary file S2. The explanation of variables

**Outcome Variable**	Delivery-related medical cost	Total direct costs during the delivery hospitalization and 6-week postpartum period
**Variable of interest**	Severe maternal morbidity	No vs. Yes (at least 1 of the 21 potentially life-threatening maternal conditions or adverse events identified by the CDC)
**Covariates**	Maternal age	< 19 years, 19–24 years, 25–29 years, 30–34 years, 35–39 years, 40–44 years, or > 45 years
	Income level	Quartile
	Type of insurance	Self-employed insured, employee insured, or medical aid
	Residential area	Seoul, metropolitan areas, small cities, or rural areas
	Mode of delivery	Spontaneous vaginal delivery, instrumental delivery, or cesarean section delivery
	Preterm birth	Delivered at < 37 vs. ≥ 37 weeks
	Parity	Nulliparous vs. multiparous
	Multiple birth status	Singleton vs. twin or more
	Adequacy of prenatal care	Adequate vs. inadequate estimated by the Kessner Adequacy of Prenatal Care Index)
	Obstetric comorbidity	0 or 1 and more assessed by Bateman’s obstetric comorbidity index)
	Delivery year	

Supplementary file S3. The codes of Severe Maternal Morbidity as defined and identified by the CDC

Severe Maternal Morbidity Indicator	Diagnosis (DX) or Procedure (PR) Code	ICD-10
**Acute Myocardial Infarction**	DX	I21.xx, I22.x
**Aneurysm**	DX	I71.xx, I79.0
**Acute Renal Failure**	DX	N17.x, O90.4
**Acute Respiratory Distress Syndrome**	DX	J80, J95.1, J95.2, J95.3, J95.82x, J96.0x, J96.2x, J96.9x, R06.03, R09.2
**Amniotic Fluid Embolism**	DX	O88.112, O88.113, O88.119, O88.12, O88.13
**Cardiac Arrest / Ventricular Fibrillation**	DX	I46.x, I49.0x
**Conversion of Cardiac Rhythm**	PR	5A12012, 5A2204Z
**Disseminated Intravascular Coagulation**	DX	D65, D68.8, D68.9, O45.002, O45.003, O45.009, O45.012, O45.013, O45.019, O45.022, O45.023, O45.029, O45.092, O45.093, O45.099, O46.002, O46.003, O46.009, O46.012, O46.013, O46.019, O46.022, O46.023, O46.029, O46.092, O46.093, O46.099, O67.0, O72.3
**Blood Transfusion***	PR	30230H0, 30230K0, 30230L0, 30230M0, 30230N0, 30230P0, 30230R0, 30230T0, 30230H1, 30230K1, 30230L1, 30230M1, 30230N1, 30230P1, 30230R1, 30230T1, 30233H0, 30233K0, 30233L0, 30233M0, 30233N0, 30233P0, 30233R0, 30233T0, 30233H1, 30233K1, 30233L1, 30233M1, 30233N1, 30233P1, 30233R1, 30233T1, 30240H0, 30240K0, 30240L0, 30240M0, 30240N0, 30240P0, 30240R0, 30240T0, 30240H1, 30240K1, 30240L1, 30240M1, 30240N1, 30240P1, 30240R1, 30240T1, 30243H0, 30243K0, 30243L0, 30243M0, 30243N0, 30243P0, 30243R0, 30243T0, 30243H1, 30243K1, 30243L1, 30243M1, 30243N1, 30243P1, 30243R1, 30243T1
**Eclampsia**	DX	O15. X
**Heart Failure / Arrest During Surgery or Procedure**	DX	I97.120, I97.121, I97.130, I97.131, I97.710, I97.711
**Puerperal Cerebrovascular Disorders**	DX	A81.2, G45.x, G46.x, G93.49, H34.0x, I60.xx, I61.xx, I62.xx, I63.00, I63.01x, I63.1xx, I63.2xx, I63.3xx, I63.4xx, I63.5xx, I63.6, I63.8x, I63.9, I65.xx, I66.xx, I67.xx, I68.xx, O22.50, O22.52, O22.53, I97.810, I97.811, I97.820, I97.821, O87.3
**Pulmonary Edema / Acute Heart Failure**	DX	I50.1, I50.20, I50.21, I50.23, I50.30, I50.31, I50.33, I50.40, I50.41, I50.43, I50.810, I50.811, I50.813, I50.814, I50.82, I50.83, I50.84, I50.89, I50.9, J81.0
**Severe Anesthesia Complications**	DX	O29.112–O29.119, O29.122–O29.129, O29.192–O29.199, O29.212–O29.219, O29.292–O29.299, O74.0, O74.1, O74.2, O74.3, O89.0x, O89.1, O89.2, T88.2XXA, T88.3XXA
**Sepsis**	DX	A32.7, A40.x, A41.x, I76, O85, O86.04, R65.20, R65.21, T81.12XA, T81.44XA
**Shock**	DX	O75.1, R57.x, T78.2XXA, T81.10XA, T81.11XA, T81.19XA, T88.6XXA

**Sickle Cell Disease With Crisis**	DX	D57.00, D57.01, D57.02, D57.211, D57.212, D57.219, D57.411, D57.412, D57.419, D57.811, D57.812, D57.819
**Air and Thrombotic Embolism**	DX	I26.x, O88.012–O88.03, O88.212–O88.23, O88.312–O88.33, O88.812–O88.83, T80.0XXA
**Hysterectomy**	PR	0UT90ZL, 0UT90ZZ, 0UT97ZL, 0UT97ZZ
**Temporary Tracheostomy**	PR	0B110F4, 0B113F4, 0B114F4
**Ventilation**	PR	5A1935Z, 5A1945Z, 5A1955Z

Source: Centers for Disease Control and Prevention, Identifying Severe Maternal Morbidity (SMM): SMM indicators and corresponding ICD codes.
